# PPI Or User Involvement: Taking stock from a service user perspective in the twenty first century

**DOI:** 10.1186/s40900-020-00211-8

**Published:** 2020-06-26

**Authors:** Peter Beresford

**Affiliations:** 1 University of Essex, , Colchester, UK; 2 Shaping Our Lives, Colchester, UK

Citizen participation, public involvement, call it what you will, is a little like the United States. Even if you willfully ignore the existence of Native Americans, the USA is hardly the ‘new world’ it is sometimes still presented as, with a history of colonization nearly half a millennium long. Similarly, provisions for public participation extend in the UK, for example, to over half a century in land use planning, while requirements in health, social care and children’s services have been in place for almost a generation [8]. Yet there is still a tendency to treat issues of participation as though they were novel and special, rather than with the seriousness that they merit as the routine part of broader policy and political frameworks they are now meant to be.

Such involvement became institutionalized as an acronym in the UK National Health Service, initially as PPI – public, patient involvement (also Patient and Public Involvement and Engagement (PPIE)) and, since 2017, as patient involvement - PI. However, public involvement is also an international development, with equivalents in Europe and the US although it is far less developed in the Global South. In the UK it is most closely associated with health and care – extending to all their aspects – from policy, practice and learning, to evaluation, research and knowledge development, but it is also an expression of a wider interest in customer involvement in the public sector more generally and of the notion of the ‘public service consumer’ [21].

I have been involved in participatory developments since 1977 as writer, researcher, educator, activist and (long term mental health) service user. I have been privileged to be part of the emerging psychiatric system survivor and disabled people’s movements over that period, as well as being involved in UK government developments at a high level, and in voluntary organizations, user led and disabled people’s organisations (ULOs and DPULOs) and activities and also international schemes, as well as initiatives in other countries. This has offered me close-up insights into theory, policy and practice developments over this period as well as opportunities to be part of them. It has introduced me to many people involved in such activities as policymakers, educators, practitioners, service users and family carers [5].

This experience has highlighted for me a range of key issues in relation to PPI – public and service user involvement and it is these I want to explore here. While I am not suggesting that this list would be shared by all concerned with these issues, the fact that I have been directly involved with many people from different standpoints, both individually and collectively, gives me some confidence that this list is likely to have at least some wider relevance and resonance. The issues that have emerged for me about public and service user involvement over more than 40 years can perhaps be best summed up in terms of a series of relationships. Issues with these relationships have characterized them so far and if we want progressive change, then we are likely to need to review and change those relationships for the future. The relationships that I have found most striking are those of PPI and involvement with:

- Broader ideology
- Politics and democracy
- Knowledge and research
- Diversity and its equalization
- Change and making change

## Ideological relations

There has long been a tendency to treat participation in isolation, to abstract it from its broader relations as if these have no bearing on its aims, nature and effect. This has been most visibly true of the tendency to ignore or understate its ideological relations, yet these predictably have had a major influence on it. As noted above, with the international shift from the 1970s to the political right, enthusiasm for the market and a reduction in state intervention, encouraged the privatization of public services and the extension of consumerist thinking into public provision. Political pressure for public involvement was framed in such terms and adopted consumerist methods of public consultation and market research. These promised public and service users a chance to be heard in public policy, but not a seat on the board. Meanwhile the pressure from service users, mobilized by the emergence of user movements and organisations, was motivated by a different pressure - for the democratization of public policy and services, especially health and welfare services which could impact heavily on their lives. Service users were primarily concerned with living their lives on more equal terms, to get the help they needed and be part of mainstream society, rather than on the nature of the service system and informing it.

One of the issues bedeviling participation and impeding its operation is the confusion that often arises between these two understandings of the idea, with service users primarily getting involved to bring about change in line with their rights and needs and state and service system calling for their involvement more narrowly to enlist their views and intelligence. The resulting confusion and conflict between involvement for empowerment and involvement for information seems to be at the heart of frequent concerns among service users that they are drawn into tokenistic and pointless participatory initiatives.

## Political relations

Another consequence of the frequent tendency to treat participation as an isolated add-on has been the failure to make broader political connections. This may have its roots in the fact that PPI has more often been examined through the lens of health and public policy than political studies and, that academia often struggles to advance interdisciplinary activity. Elsewhere, I have charted four stages in the development of western representative democracy and the emergence of provisions for participation. The timescales provided relate to the UK and can vary with different countries. Thus:

- Working for universal suffrage in representative democracy and the achievement of social rights, like the right to decent housing, education and health, from the late nineteenth to mid twentieth century;
- Provisions for participatory democracy and community development, associated with the 1960s and 70s
- Specific provisions for participation in health and social care, from the1980s through to the first decade of the twenty first century
- State reaction and service user-led renewal as conflicts and competing agendas develop, from 2010 onwards [6].

The third stage identified approximates to UK developments like the setting up of the National Institute for Health Research INVOLVE and increasing requirements for PPI in research and provision. In the fourth stage, however, we can also see the increasing articulation of rifts between state/service system-led ideas of PPI and consumer involvement and the pressure from service users and their allies for more say and democratic control over their lives and services and a questioning of formal arrangements, as they increasingly identified these as inadequate for their purpose ([16]; Madden and Speed [4, 6, 10, 18];).

The period since 2010 has been one of the strengthening of right wing neoliberal politics, with an even stronger shift during the latter part of that era to populist right wing politics. This has conspicuously developed in the US, parts of Europe and the UK. While such politics are associated with demagoguery and xenophobia, they are also linked with attacks on public services, cuts in public service budgets and the political stereotyping and ‘othering’ of some groups. This tends to be at increasing odds with service user pressure for involvement and change, especially since service users, notably disabled people and mental health service users, have become particular targets. Significantly in the UK such activity has been associated with an extreme ‘welfare reform’ policy which has restricted eligibility for both disability and employment benefits. Scapegoated those receiving them, it has been associated with little if any PPI or user involvement [22]. Dissatisfaction with promises of user involvement have become more explicit [20]. Conflicts between the two competing understandings of involvement have become both more explicit and more contested, with radical new user groupings and organisations emerging like Disabled People Against Cuts, Spartacus and the Mental Health Resistance Network, which have been dismissive of traditional consultative involvement while in the vanguard of opposing welfare reform, especially using direct action [2]. This has also led to distinctions increasingly being drawn between PPI and user led activities and involvement, with the former treated with rising wariness, suspicion and hostility. Thus one critique dismisses PPI as a ‘zombie’ policy, unproductive for change and improvement [17].

There is an increasing sense that service user-led research and service users in research face discrimination and an uphill struggle for equality and are still operating on a far from level playing field. This is seen to extend to the allocation of research grants, the focus of research, support for user-controlled research, the value attached to emancipatory research and career opportunities for user researchers, all of which it is argued are inferior [11, 23, 24]. At the same time there has been a growing sense among service users and their organisations that shared language but different meanings and intentions has led to the widespread subversion of their ideas and innovations in the service system, with notions like ‘self-management’ ‘peer support’, ‘peer worker’ and person-centred support, all being undermined and reframed in terms of dominant understandings and values [5, 6, 24].

## Relations with knowledge

The other, no less important relationship here, has been with research and knowledge development. Research has always been an important locus for involvement, right from the earliest days of the UK disabled people’s movement. This is because of its role as a key source of knowledge. Traditional positivist research has emphasised values of neutrality, objectivity and distance. Service users and their organisations, however, quickly challenged this. They have questioned the ‘unbiased value-free’ position, based on professional expertise of the researcher which has been seen as a central tenet of such research.

Central to this is its introduction of and valuing of what has come to be called experiential knowledge; that is to say knowledge based on people’s subjective and lived experience, rather than solely professional training, accreditation or research and experiment. However, such experiential knowledge has tended to be granted less value and credibility under the operation of traditional research values and principles. However, the knowledge claims of researchers without such direct experience are still seen to be stronger.

This persists with belief in randomised controlled trials and systematic reviews as the ‘gold standard’ of research.

However service users have turned these arguments on their head, arguing that by devaluing experiential knowledge we lose a key knowledge source. What this also means crucially is that if an individual has direct, lived experience of problems like disability or poverty, or of oppression and discrimination, of cuts and ‘austerity’, of racism and sexism, when such traditional positivist research values are accepted, what they say – their accounts and narratives - will be seen as having less legitimacy and authority. Because people experiencing hardship will be seen as ‘close to the problem’, they cannot claim they are ‘neutral’, ‘objective’ or ‘distant’ from it. So, in addition to any discrimination and oppression they already experience, they are likely to be seen as a less reliable and a less valid source of knowledge. By this logic, they can also expect routinely to face further discrimination and be further marginalised [1]. This issue of marginalising the knowledge of particular vulnerable groups has begun to be talked about in terms of ‘epistemic violence’ and ‘epistemic injustice’ [12, 15], meaning devaluing and marginalising the knowledges of people who suffer abuse, discrimination and oppression. There is now rising pressure for epistemic *justice* - for ensuring that everybody can contribute to creating a general knowledge base and that perspectives of entire social groups are no longer excluded from that process. While we have begun to see the real involvement of ordinary and disadvantaged people in research, for example people with learning difficulties, who communicate differently or experience dementia, we also seem to be seeing some retreat from this engagement [19] We have also begun to hear how service user researchers may not only feel marginalised in research structures, but also by other service users [11].

## Relations of exclusion

A longstanding problem associated with participatory initiatives is their failure to be inclusive of diversity. The traditional methods first used in land use planning gave early warning of this, usually generating a narrow response of white, middle class, middle aged men [7]. Since then both service users and their organisations, as well as formal and state-led initiatives have struggled to be more inclusive and address diversity with greater equality. However, a large scale research and development project supported by the UK Department of Health, carried out by the user led organization (ULO) Shaping Our Lives, in 2013 identified a wide range of exclusions that seem to continue to operate in relation to efforts to involve people. This identified five key groups of people as service users who are excluded, on the basis of:

- Equality issues; in relation to gender, sexuality, race, class, culture, belief, age, impairment and more;
- Where people live; if they are homeless, in prison, in welfare institutions, refugees and so on;
- Communicating differently; if they do not speak the prevailing language, it is not their first language, they are (D)deaf, use sign language or are non-verbal;
- The nature of their impairments; when these are seen as too complex or severe to mean they could or would want to contribute;
- Where they are seen as ‘unwanted’ voices; they do not necessarily say what authorities want to hear, are seen as a problem, disruptive etc., including for example, people who identify as neuro-diverse, with dementia, etc. [3].

The research emphasised the need for out-reach approaches to involvement drawing on community development techniques. Recently, The Covid-19 pandemic has also highlighted the need to develop further imaginative ’non-contact’ forms of involvement to reduce such inequalities.

The discriminatory and exclusionary relations Shaping Our Lives identified can operate both locally and internationally. Broader issues relating to exclusion are also regularly identified. These relate both to the tendency to marginalise indigenous populations, both in mainstream life and in compensatory arrangements, like schemes for participation. These can apply to Black and minority ethnic and majority ethnic populations. It is becoming apparent that western concepts like ‘user led’ research may have limited relevance in the Southern Hemisphere and indeed that work on user involvement more generally is both different and less developed in other regions [13] In the UK, the ‘*Still We Rise*’ project is creating an archive of the work done by African, African Caribbean, and Asian users and survivors in the UK, ‘given that existing archives have limited material relating to this history’ [14].

## Relations with making change

As has already been said, from this author’s experience, while some service users whose opinions have never been sought before can value information seeking exercises, the primary reason that people get involved is in order to make change [8]. I have been involved in research with people with life-limiting conditions, close to the end of their life, where they have said that they realize that their involvement will come too late to change things for them, but they are doing it for others. It may not have been measured, but the altruistic impulse seems to loom large in people’s motivation to get involved. That is why some service users are frustrated and confused by a consumerist approach to involvement, although, to be truthful, there may have been no suggestion that their views would be the defining ones and the subtext may have clearly been that the aim was just to find out what they thought and acting on it no more than a possibility, given other viewpoints and imperatives that might be being taken into account. Thus the association of such a market-based model of involvement by some service users with ‘extraction’ of their knowledge, rather than with their personal and political *empowerment* – which tend to be their implicit goals.

It is also important to be aware that when we are thinking of change in relation to participation, that not all change may be seen by service users as in their interests. Instead the purpose may be to inform service providers’ own revision of *their* priorities, *their* budgetary decisions in making cuts in services and support and in adding to *their* knowledge base, rather than accessing and prioritizing service users’ experiential knowledge. People get involved to bring about change in line with their rights and needs; what they and their peers want, not merely for prevailing agendas. Thus, much mental health research by pharmaceutical companies, understandably, is focused on developing new drugs or drug variants – ‘me too’ drugs. But we know that mental health service users are much more interested in holistic, more social model-based responses to their distress [9]. Thus their involvement may actually have counter-productive consequences for them and instead of advancing their knowledge, may just extend that of ‘big pharma’.

One of the comments made in feedback to this article from the Journal’s reviewers was that it needs to be made clear that it offers a personal view. I am happy for that to be clear. But it is a personal view shared by many disabled people and service users, particularly service user researchers and evidence they have generated. And one of the underpinning points that they have long made about research is that it is universally affected by ‘personal opinion’, in the selection of focus, research question, methodology, conclusions and relationship with research participants. This is a key point raised by years of user research from the very first time, that disabled people challenged mainstream researchers. I have found many of the three reviewers’ comments helpful. My thanks to them. PPI continues to be a contentious issue without any consensus, where how we see things does depend significantly on our personal perspective. We *all* need to remember that.

## Conclusion

It is important to be clear about the obstacles in the way of effective PPI and user involvement because reducing and removing them offers ways forward for the future. These can also be spelled out in terms of a range of positive actions that need to be taken. These include:

- More inclusive methods of involvement need to be employed, crucially moving beyond continuing reliance on contribution through conventional meeting and written forms, instead reaching out to people, their organisations and communities, rather than relying on them responding to calls.
- Funders and the funding system need to improve and equalize the funding they offer user led organisations. These provide crucial platforms for developing user involvement and co-production, co-learning, user led services and support, and user controlled research.
- The United Nations Convention on the Rights of Persons with Disabilities (UNCRPD) sets out a model for ‘independent living’ derived from the disabled people’s movement which provides an invaluable and unifying philosophical base to underpin any effort to involve service users.
- Increasingly service user and their allies draw a distinction between PPI and their calls for greater involvement and empowerment in research. Such a gap needs to be carefully examined and challenged.
- A thoroughgoing enquiry is needed into the practical and methodological implications of user involvement and experiential knowledge in research through from research teaching and learning, developing participatory PhDs to ensuring parity in research policy, institutions and practice.
- Research careers for service user researchers need to be further resourced and developed, rather than them hived off and restricted to narrow roles of ‘peer researchers’.

## Data Availability

Not applicable
